# Non-pharmacological Radical Methods for Treating Postpartum Depression Around the Globe: A Narrative Review

**DOI:** 10.7759/cureus.76052

**Published:** 2024-12-20

**Authors:** Sushmitha G, Anantha Eashwar V M, Sujitha Pandian, Monica Albert Sekhar, Shirley Esther Pricilla

**Affiliations:** 1 Preventive Medicine, Sree Balaji Medical College and Hospital, Chennai, IND

**Keywords:** affective disorders, cognitive behavior therapy, holistic health, maternal-child health, mental health, psychotherapy

## Abstract

Mental health conditions during pregnancy, especially postpartum depression (PPD), can have profound and long-lasting effects on the individual, impeding her ability to bond with her child and disrupting the family dynamics. Although pharmacological treatments like antidepressants are the mainstay treatment options, several mothers have concerns about their safety and potential side effects, especially breastfeeding mothers. There is an emerging interest in exploring the use of non-pharmacological interventions as an alternative treatment modality for PPD. This review focuses on the effectiveness of non-pharmacological options like cognitive behavioral therapy, interpersonal therapy, the use of mobile games, technological interventions, and creative art techniques. This review also highlights the existing gaps like the dearth of research from lower socioeconomic countries where postpartum women face several barriers in accessing the much-needed support as stigma surrounding mental health still exists and the lack of studies to assess the long-term effects of these interventions.

## Introduction and background

Postpartum depression (PPD) proves to be a significant mental health concern as it continues to be underdiagnosed, underreported, and overlooked even in the modern era [1]. The prevalence of PPD ranges from 40% in lower socioeconomic countries to 9% in higher socioeconomic countries [2]. If left untreated, depressive symptoms can be detrimental for both mother and child. PPD occurs within four weeks to one year post-delivery. Symptoms could range from a depressed mood, lack of interest in activities that were previously enjoyed, changes in appetite, and difficulty falling asleep or sleeping more than usual. She may also present with fatigue, difficulty thinking or concentrating, trouble making decisions, constant thoughts of death or suicide, bouts of crying without reason, decreased interest in the baby, and feelings of not being bonded with the baby. To clinically diagnose PPD, five symptoms along with anhedonia or depressed mood, must be present every day for two weeks [3,4]. The recent edition of the Diagnostic and Statistical Manual of Mental Disorders (DSM-5) proposed a change where PPD is now categorized as “depressive disorder with peripartum onset” [5].

Treatment includes pharmacologic and non-pharmacologic modalities [6,7]. Although antidepressants are a recognized modality of treatment, many mothers have concerns about their safety, especially breastfeeding mothers. Several doubts and misinformation surrounding antidepressants have deterred mothers from utilizing them. Non-pharmacological treatments utilizing psychotherapy like cognitive behavioral therapy and interpersonal therapy are well received by the mothers [8].

## Review

Methodology

A thorough search of the internet databases, including PubMed, Scorpus, and Web of Science was done to conduct the narrative review. All the relevant articles were searched using the keywords "Postpartum depression," “Postnatal depression,” “Non- pharmacological methods,” “Cognitive Behavioral Therapy,” “Psychotherapy,” “Interpersonal Psychotherapy,” “IPT,” “Digital mental health,” “Peer group support,” “Exercise,” "Acupuncture,” “Infant massage,” “Newborn massage,” “Transcranial Magnetic Stimulation,” and “Light Therapy”. The inclusion criteria included articles that were published in peer-reviewed publications, written in English, and focused on PPD and non-pharmacological treatment. Articles published between the years 2000 and 2024 were included in the study. Further, the authors conducted manual searches to include other important studies. Original research papers, systematic reviews, meta-analyses, and RCTs were chosen for inclusion. The articles that focused on research conducted on non-pharmacological methods for the treatment of PPD were included in the narrative review. The rest of them were excluded. The exclusion criteria included any duplicate articles, articles published in non-peer-reviewed journals or written in a language other than English, and those with missing texts. Articles that fit into the inclusion criteria were only chosen and screened during selection. The authors reached an agreement through discussion to resolve any differences regarding choosing articles according to the inclusion criteria.

Behavioral activation gaming app for treating depression

A pilot study evaluated the one-of-a-kind gaming app called “The Guardians”, a behavioral activation (BA) mobile game, which was designed to target pregnant women with elevated depressive symptoms [9]. This was based on the theory that depressed individuals tend to be isolated and have an avoidant nature, which worsens the depressive symptoms [10]. The game was a single-arm trial that had participants complete biweekly assessments of depression and anxiety for a period of 10 weeks. The Patient Health Questionnaire (PHQ-9) and the Generalised Anxiety Disorder-7 were used to measure their symptoms. The player had to complete a set of tasks each day, which included participating in daily activities. The players can choose from a list of real-life activities and can log their own activities. The study found that users found the app feasible and acceptable to the mothers. Participants reported that it effectively encouraged them to engage in mood-enhancing activities and decreased their depressive symptom scores over the trial. This shows that mobile games, designed to cater to mothers, can be tried as a new modality to treat depressive symptoms [9].

Effectiveness of cognitive behavioral therapy in combating postpartum depression

CBT is a structured, therapeutic approach given for a short time with the goal of helping individuals recognize and change their maladaptive behaviors and thought patterns that contribute to their depression. CBT combines cognitive restructuring and psycho-education behavioral changes to encourage healthier, balanced thought patterns and formulate coping strategies [11,12]. An interventional study conducted by Chungu et al., based in Zambia, showed that the group of postnatal mothers diagnosed with PPD who received CBT via telephone from trained psychologists had decreased scores on the Edinburgh Postnatal Depression Scale (EPDS), which is the most commonly used scale for assessing PPD [13]. Telephonic CBT in lower socioeconomic countries is a great way of reaching people with little access to mental health care and awareness of PPD. A RCT done by Fuhr et al., in India, utilized the help of peers in delivering the CBT [14]. The peers were laywomen from the same locality and were trained in delivering CBT. The findings showed that there was a moderate reduction in depressive symptoms and highlighted it as a cost-effective alternative. More studies like these can be modified for cultural appropriateness and utilized in lower socioeconomic countries. 

Happiness, Understanding, Giving, and Sharing Intervention

Happiness, understanding, giving, and sharing (HUGS) is a programs that help improve mother-child bonding in mothers diagnosed with PPD, as previous research states that maternal depression hampers mother-child bonding. This intervention goes one step further and teaches vital skills to the mothers and how to bond again. Session one teaches the mother how to play with the infant, the importance of physical contact, how to identify deficits, and how to find solutions accordingly. Session two emphasized observing and understanding the baby’s signals and motivated positive interactions through guided exercises. In session three, parents learned to respond to infant cues and overcome any negative thoughts. Finally, session four served as a reinforcement session about the benefits of mother-child bonding in the development of infant cognition. The successful treatment of PPD lies in early detection, so combining CBT in healthcare will be an added benefit, as it helps in identifying mothers who are at risk of developing PPD [15].

Interpersonal psychotherapy for reducing depressive symptoms

Interpersonal psychotherapy's (IPT) main focus lies in improving interpersonal relationships. The effectiveness of IPT has been studied in the treatment of depression, anxiety disorders, and post-traumatic stress disorders [16]. Because of the flexibility of IPT, it can be given in various settings like hospitals, community-based, and even given individually. It is centered on the principle that the relationships one holds and the life events one has experienced influence the mood. The four problem areas that IPT focuses on are grief, life changes, isolation, and interpersonal conflicts. There are three phases of IPT. The initial phase focuses on building a rapport, taking the full history, and forming a diagnosis by the therapist. The therapist then understands the problem areas and formulates a working plan. The middle and the termination phases have several sessions conducted by the therapist. One specific interpersonal area is chosen and worked on, and the therapist helps the patient address the challenges in healthier ways [17]. A systematic review conducted by Kang et al. found that when IPT was given to depressed mothers, it led to a decrease in the EPDS score after the completion of treatment, as well as a positive adaptation to marriage [17]. Another systematic review conducted by Xang et al. found that when IPT was given for more than 12 weeks to depressed mothers, it resulted in decreased EPDS scores and increased satisfaction with the family [18]. However, the study did not find significant differences in social support or mother-child bonding, as the papers included in the review were small. This shows that IPT is a valuable tool for treating PPD and must be actively incorporated into mental health programs by the governments.

Peer support in alleviating depression

In a qualitative study, a group of women enrolled in peer support groups was selected. It was found that the peer support projects made a significant impact on new mothers who were downtrodden, had faced domestic abuse, were not accepted back into their maternal homes, were refugees, or had multidimensional social support needs. Practical solutions were provided to the new mothers, and help was even offered in taking care of the baby or accompanying them to the supermarket to purchase items [19]. This study underscores the pertinent role of social support on maternal mental well-being.

Use of technology in combating postpartum depression

Internet-Based Intervention: Netmums and PandaMom

An RCT conducted in the UK, called Netmums, introduced a 12-session online module to participants who had scored higher on the EPDS [20]. The module consisted of topics like depression, motherhood, and childcare. The participants had to finish each module, and trained professionals called the participants weekly and cleared their doubts. The postpartum mothers were even allowed to meet other mothers in the RCT, to get the much-needed peer support. The study had positive improvements in depressive symptoms in women who had good adherence.

Another interventional study conducted by Schmidt JH et al. in Germany, called PandaMom, was based on the principle of CBT but was delivered via a self-help online program [21]. The eligible participants, after registering online, had access to the program. This program, along with educational modules, self-report questionnaires, and workbooks, also included exercise videos. Another unique aspect of this program lies in its customization. According to the participants' responses, tailor-made feedback was given by a team of psychologists. Interventions like these set the stage for more such future research in the field of PPD leveraging technology and finding innovative solutions [21].

Digital Mental Health Intervention and the Role of Husbands

In a mixed-method study conducted by Bhardwaj A et al. in Nepal, which recruited mothers who scored more than nine on the PHQ-9, android phones, and smartwatches were used to track physical activity, such as walking or vehicle use [22]. The data from GPS was used to measure activity frequency. As depressed mothers will show less consistent physical activity than non-depressed mothers, all the mothers were strictly monitored. Depressed mothers will be guided to incorporate regular mood-enhancing physical activities and will be asked to set a specific goal, for example leaving the baby with any family member and visiting the market. The trained counselors ensured that the mothers completed their goals. Next, a Bluetooth beacon was placed on the infant’s clothing, which was used to measure the proximity of the mother through signals received via the mobile phone. This measured how much time the mother and child spent together, as previous studies show that depressed mothers are devoid of social support and spend more time exclusively with their children [22]. A mother who has her own routine, with family members helping her with childcare, is associated with a lower risk of PPD [23]. The utilization of the mobile app “StandStrong” played a crucial role in this study by visualizing data [24]. Using this data, counselors monitored activity and used these insights during therapy sessions. Additional app features included direct messaging to the counselors, educational materials, and awards for meeting activity goals. Another unique angle that this study provided was making the husbands participate in the study. Qualitative interviews were done with the husbands, and themes were identified. This was then used to counsel the husbands on equitable gender norms, changing the attitudes surrounding child care, and social support. At the end of the study, the mean depression score had lowered in the women whose husbands had a positive attitude toward childcare and social support [22]. This shows that more husbands must be interviewed and counseled regarding childcare, as interventions are usually targeted toward the mother, rather than the family as a whole.

Complementary and alternative medicine in postpartum depression

Effectiveness of Exercise-Based Interventions in Preventing Postpartum Depression

An RCT done by Lewis BA et al. recruited postpartum women who had a history of depression [25]. The study had three groups, and the mothers were recruited into either a telephone-based exercise intervention, a telephone-based wellness/support intervention, or usual care, with each lasting six months. The intervention's main aim was to reduce barriers like childcare, travel costs, and time, which often hinder face-to-face interventions, while still offering one-on-one counseling. At the end of the study, participants recruited in the exercise group reported decreased symptoms of depression when compared to participants in the wellness group. Another meta-analysis found that moderate-intensity aerobic exercises performed for 35-45 mins had a more significant effect on depression scores compared to low-intensity and high-intensity aerobic exercises. However, the study noted that the difference could also be influenced by team-based activities or supervised exercises [26]. Further research into this could bring about positive changes for postpartum mothers globally.

Creative Art Therapy

It is a treatment that incorporates creative and artistic activities to support emotional and psychological well-being. This therapy encourages patients to use creative expression to address their emotional difficulties. "Creativity" in creative art therapy (CAT) is the process where individuals explore their subconscious through artistic activities, increasing their ability to express emotions and stimulating their thinking. "Artistic quality," on the other hand, refers to the enjoyment derived from engaging in the creative process, which can help decrease stress [27].

It encompasses a wide range of artistic activities, including singing, painting, dancing, sculpture, drama, film, and poetry. These activities give patients an opportunity to engage with their emotions and help them express themselves in a non-verbal, yet deeply therapeutic, manner. Since the activities are creative and do not involve taking any medications, they are more easily accepted by patients and their families, which indirectly increases treatment compliance. Additionally, CAT can be conducted independently by the patient, offering a sense of privacy and autonomy, which is an advantage for those who may be hesitant to seek traditional therapy.

Despite its potential, the application of CAT in clinical settings remains in the early stages, especially in the context of PPD. Several medical centers worldwide are beginning to acknowledge the importance of mental well-being in postpartum women and are exploring the use of CAT as a tool for improving mental health outcomes. However, there is still a lack of large-scale studies to confirm the efficacy of CAT in treating PPD. A recent study done by Xu et al. found that the intervention group showed significantly better results than the control group [28].

Yoga as an Interventional Tool for Postpartum Depression

A randomized control study, done by Butner MM, examined the effect of Gentle Vinyasa Flow yoga on postpartum women with a score of more than 12 on the Hamilton Depression Rating Scale (HDRS) [29]. The yoga intervention was taught for eight weeks, consisting of 16 classes, by a certified yoga instructor. At the end of eight weeks, the women who had received yoga as an intervention showed improvement in their depression scores. Additionally, it was also observed that women in the yoga group also improved in their anxiety scores.

Acupuncture in Postpartum Depression

Acupuncture, a traditional Chinese medicine, has been demonstrated to be an effective alternative form of treatment for depressive disorders. However, its role in PPD remains controversial. A systematic review and meta-analysis done by Li et al. revealed that acupuncture treatment of postpartum women with depression significantly reduced the HDRS scores [30]. However, the analysis did not find significant effects of acupuncture on other scales of depression like the Edinburgh Postpartum Depression Scale (EPDS), or in serum estradiol levels.

Mother-Infant Massage Classes

A systematic review found that infant massage classes, guided by a trained instructor, can improve bonding and alleviate depressive symptoms [31]. The review also found that these classes also provided an opportunity for the mothers to interact with each other, and thus the role of peer group support comes into play. Low levels of self-confidence in new mothers are common, and mothers reported that after attending these classes their confidence improved. A qualitative study done by Midtsund et al. found that mother-led infant massage classes improved the quality of time a mother spent with her baby, and her attachment to the baby grew as they massaged the baby and maintained eye contact with them [32]. Mindell et al., in their study, noted that infants who were massaged had better sleep at night [33]. Mother-led infant massage classes may be a cost-effective solution and help in decreasing depressive symptoms.

Physical Therapy for Combating Postpartum Depression

Transcranial magnetic stimulation (TMS) is one of the non-invasive methods for treating PPD but with limited data on efficacy and safety [34]. An open-label pilot study on the use of TMS in treating major depressive disorder (MDD) was carried out by Kim DR et al., in which pregnant women in the latter half of the pregnancy were subjected to 20 sessions of 1-Hz TMS at 100% of motor threshold (MT) to the right dorsolateral prefrontal cortex with a total study dose of 6000 pulses [35]. Seven of the ten participants who received TMS showed a marked reduction in depression scores with no harm to the fetus [36]. Larger longitudinal studies are needed to understand the efficacy of TMS in treating PPD. If found to be beneficial, this can be one of the methods used in treating MDD among women who do not wish to opt for pharmacological treatment. 

Light Therapy

Light therapy involves exposure to a device that emits light at a certain intensity. When used among patients with depression, it stimulates the intrinsic photosensitive retinal ganglion cells to improve depressive mood and sleep [37]. An open trial of using light therapy to treat PPD was first conducted in 2002 among 16 pregnant women with MDD, in which there were significant improvements in depressive symptoms following the intervention [38]. Though several studies were conducted to check the efficacy of the same in treating PPD, either their sample sizes were small or the findings were not consistent [39,40]. A meta-analysis by Li X et al, on using light therapy for treating PPD, found that light therapy is effective for treating pregnant women with depression and sleep problems [41].

Tackling PPD needs a multifaceted approach. Governments need to prioritize implementing non-pharmacological interventions within healthcare systems and bring about policy changes. While many high-income countries have established supportive policies in their system such as paid maternity leave and better child care, these still remain a distant reality in lower-income countries. Measures like these are in need of the hour, as the absence of such policies is also a major factor for maternal mental health problems [42].

The characteristics of the studies discussed in the review are summarized in Table 1.

**Table 1:** Summary of non-pharmacological interventions used in treating PPD BA: behavioral activation; PPD: postpartum depression; RCT: randomized controlled trial; CBT: cognitive behavioral therapy; IPT: interpersonal therapy; PHQ-9: Patient Health Questionnaire-9; GAD-7: Generalised Anxiety Disorder; EPDS: Edinburgh Postpartum Depression Scale;  ERA: Parent-Child Early Relational Assessment; PSI: Parenting Stress Index; BDI: Beck Depression Inventory; DASS-P: Depression Anxiety Stress Scales, CGI-S: Clinical Global Impression Severity, HDRS-17: Hamilton Depression Rating Scale; CAT: creative art therapy; TMS: transcranial magnetic stimulation

S.No	Study	Study design	Study population	Sample size	Intervention used	Duration of intervention	Result
1.	Vanderkruik et al., 2024 [9]	Single arm intervention pilot study	Antenatal women	n=18	BA gaming app	10 weeks	Changes in PHQ-9 scores: There was a significant decrease in the weekly scores as the participants spent more weeks in the study and completing the daily activities given in The Guardians app. Changes in GAD-7 score: The results showed that there was a reduction in anxiety scores starting from the fourth week onwards
2.	Chungu HW, 2017 [13]	RCT	Postpartum women	n=64	CBT	12 sessions on a weekly basis	The group that received both CBT and pharmacotherapy showed a significant reduction in depression scores
3.	Fuhr et al., 2019 [14]	RCT	Postpartum women	n=280	CBT	14 sessions divided into four phases starting from the second or third trimester till 6 months after childbirth	The intervention group showed a moderate reduction in PHQ-9 scores than the control group and also found this to be a cost-effective treatment modality
4.	Holt et al., 2021 [15]	RCT	Postpartum women	n=77	CBT	Nine sessions on a weekly basis focusing on PPD, three couple sessions, and an extra four sessions targeting the mother-child bonding	The outcome of the intervention group was measured using ERA, PSI, and BDI. There were no immediate changes in both scores post-intervention. However, the six-month follow-up showed that participants in the intervention group had much better bonding with their infants. There were no differences in the PSI scores, while a significant reduction in BDI was observed
5	Kang et al., 2024 [17]	Systematic review	Postpartum women	n=4	IPT	12-16 weekly sessions	The four studies chosen were RCTs. The studies showed that depression scores had reduced significantly in the group, which had IPT as an intervention
6	Wang et al., 2023 [18]	Systematic review	Postpartum women	n=9	IPT	14-12 weekly sessions	It was found that IPT given for four to eight weeks had the highest impact on depression scores. IPT given for more than 12 weeks had a significant increase in satisfaction with the family
7.	McLeish et al., 2017 [19]	Qualitative study	Postpartum women	n=47	Peer support	Starts from pregnancy and goes for a maximum of two years postnatal	Peer groups helped participants alleviate depressive symptoms and improved their confidence in childcare. Participants also felt that their stress levels decreased after receiving practical support from peer supporters
8.	O’Mahen et al., 2014 [20]	RCT	Postpartum women	n=83	Internet-based BA	12 online module sessions and 12 telephone support sessions	The intervention group showed significant reduction in both EPDS and GAD-7 scores. However, there was no significant difference in self-reported bonding or perceived social support
9	Schmidt et al., 2024 [21]	Longitudinal pilot trial	Antenatal women	n=149	Guided self-help online program based on IPT and CBT	The program consisted of two phases. The first phase focused on the antenatal period, and the second phase started two weeks after giving birth. It consisted of six basic and four supplementary modules	The results show that there is a mean decrease in the scores of EPDS and DASS-P in those who completed the program. But the adherence to completion of modules was very low (n=40)
10.	Bhardwaj et al., 2024 [22]	Mixed method	Postpartum women and their husbands	n=24	Digital mental health intervention	The data that was collected from the smartphone/smartwatch and from the beacon attached to the infant was used to give customized counseling sessions for both the postpartum women and their husbands	At the end of the study, the mean score of depression was lowered in those women whose husbands had a positive attitude toward childcare and social support
11.	Lewis et al., 2021 [25]	RCT	Postpartum women	n=450	Exercise-based intervention	Telephone-based exercise intervention, telephone-based wellness/support intervention, and usual care	Participants recruited in the exercise group reported decreased symptoms of depression in EPDS when compared to participants of the wellness group
12.	Xu et al., 2023 [26]	Meta-analysis	Postpartum women	n=26	Exercise-based intervention	Three subgroups were identified: Individual exercise, group-based exercise, and unsupervised exercise, which ranged from 20 minutes to 60 minutes	The study found that moderate-intensity aerobic exercises performed for 35-45 minutes had a more significant effect on depression scores compared to low intensity and high-intensity aerobic exercises
13.	Xu et al., 2024 [28]	Systematic review and meta-analysis	Postpartum women	n=12, which comprised of only RCTs	CAT	-	It was found that the intervention group showed significant improvement in their depressive symptoms compared to the control group
14.	Buttner et al., 2015 [29]	RCT	Postpartum women	n=57	Yoga	16 classes over eight weeks	At the end of eight weeks, the women who had received yoga as an intervention showed improvement in their depression scores. Additionally, it was also observed that women in the yoga group also improved their anxiety scores
15.	Li et al., 2019 [30]	Systematic review and meta-analysis	Postpartum women	n=8, which comprised of only RCTs	Acupuncture	-	Acupuncture treatment significantly reduced the HDRS. However, the analysis did not find significant effects of acupuncture on other scales of depression like EPDS or in serum estradiol levels
16.	Geary et al., 2023 [31]	Systematic review	Postpartum women	n=8	Mother-led infant massages	The massages were given for two weeks to three months post-delivery	Significant reduction in EPDS scores was noted. The intervention also calmed the mothers and helped them bond with their infants. The mothers also learned how to interpret their infants' cues
17.	Midtsund et al., 2019 [32]	Qualitative study	Postpartum women	n=12	Mother-led infant massages	six weeks on a daily basis	The participants felt that the mother-led infant massage class improved their self-esteem and helped them connect with their babies
18.	Mindell et al., 2018 [33]	RCT	Postpartum women and their infants till 18 months old	n=123	Mother-led infant massages	two weeks on a daily basis	Improved mother and infant sleep quality was noted in the intervention group
19.	Lee et al., 2021 [34]	Systematic review and meta-analysis	Antenatal women and postpartum women up to one year after childbirth	n=10	TMS	The sessions ranged from 1-77	There was a significant reduction in depressive symptoms post the intervention. Side effects like headache and pain at the stimulation site were noted in the antenatal women. Side effects in infants included preterm birth
20.	Kim et al., 2011 [35]	Pilot study	Antenatal women from 14-34 weeks of gestation	n=10	TMS	four weeks	Post the intervention, there was a mean decrease of 60% in the scores of HDRS-17. The mean CGI-S and BDI scores showed a significant decrease in the mean values. The maternal adverse effects noted were hypotension and headaches. Fetal heart monitoring was done before, during, and after the sessions. It showed normal readings, and the infants were all healthy at the time of delivery
21.	Oren et al., 2002 [38]	An open trial	Antenatal women	n=18	Light therapy	60 minutes daily for five weeks	Moderate improvement of depressive symptoms was noted post light therapy. After the withdrawal of treatment, there was an increase in the depressive symptoms
22.	Corral et al., 2007 [39]	Single arm trial	Postpartum women	n=15	Light therapy	six weeks	Initial improvement of EPDS scores were noted post-treatment. Depression scores were higher after withdrawal
23.	Li et al., 2023 [41]	Systematic review and meta-analysis	Postpartum women	n=8	Light therapy	Three-six weeks	It was noted that light therapy was effective for treating pregnant women and postpartum women with depression and sleep problems. It was also found that a shorter duration of therapy led to better improvement in depressive symptoms

## Conclusions

In conclusion, PPD poses a significant mental health challenge for new mothers, with debilitating long-term effects on both maternal well-being and their ability to bond with their children. While pharmacological treatments like antidepressants remain the standard mode of treatment, non-pharmacological interventions are slowly emerging as promising alternatives as they address the concerns of side effects, especially for breastfeeding mothers. The current review has highlighted various effective non-pharmacological treatments, such as CBT, IPT, mobile-based interventions, CAT, and even complementary approaches like yoga and acupuncture.

These therapies have proven to be beneficial in alleviating depressive symptoms and also foster positive changes in maternal behavior and bonding. However, despite their huge potential, there remain gaps in accessibility, customized treatment solutions, and large-scale research. Future research should focus on optimizing these interventions, conducting trials, and publishing the results. Governmental and non-governmental agencies must fund trials and must ensure they are easily accessible to the mothers, advocated to the entire family, culturally appropriate, and must be integrated into routine maternal health care programs. More focus on attitudes toward parenting must be brought about in lower socioeconomic countries. It is also imperative that healthcare systems worldwide adopt a holistic approach to treating PPD, incorporating both pharmacological and non-pharmacological treatments. This approach will lay a foundation for good maternal mental health enhancing infant development and improving the overall family dynamics.
